# IDENTIFYING PREDICTORS OF SURVIVAL TO 100 IN OKLAHOMA USING CENTENARIAN BIOGRAPHIES

**DOI:** 10.1093/geroni/igz038.2646

**Published:** 2019-11-08

**Authors:** Nadia Firdauysa, Jyoti Bhatta, Alex J Bishop

**Affiliations:** Oklahoma State University, Stillwater, Oklahoma, United States

## Abstract

The purpose of this study was to identify key predictors of centenarian survival in Oklahoma. Data originated from N = 607 centenarian biographies maintained within Oklahoma Centenarians, Inc. historical records database. Biographies were analyzed and coded for demographic content. IBM/SPSS 23.0 was then used to compute linear regression analyses to examine the association of predictor variables sex, race, education, cohort, and longevity secret relative to days of survival. Only race (std. B = .10, p < .05) and cohort (std. B = -.11, p < .01) emerged as significant predictors of overall survivorship. Non-white centenarians live longer than their White-Caucasian counterparts; whereas earlier-born cohorts have shorter survival. Closer examination of these findings revealed that non-White centenarians have historically lived an average of 300.38 days longer than their White counterparts; whereas later born cohorts have historically an average of 48.10 days shorter than earlier-born cohorts. Despite the fact that sex and subjective longevity secrets failed to yield any significance, further inspection revealed two interesting highlights. First, centenarian males have historically lived an average of 147.89 days less than female centenarians. Second, centenarians who cite God as the secrete to their longevity have historically lived 100.23 fewer days than centenarians who attribute their longevity to something else. Results have implications to further understanding the interplay of race and human longevity, as well as variables attributed to improved survivorship across successive cohorts. Further discussion relative to health practices and policies to improve longevity in states like Oklahoma will be further highlighted.

